# Predicting functional outcomes of patients with spontaneous intracerebral hemorrhage based on explainable machine learning models: a multicenter retrospective study

**DOI:** 10.3389/fneur.2024.1494934

**Published:** 2025-01-10

**Authors:** Bin Pan, Fengda Li, Chuanghong Liu, Zeyi Li, Chengfa Sun, Kaijian Xia, Hong Xu, Gang Kong, Longyuan Gu, Kaiyuan Cheng

**Affiliations:** ^1^Department of Emergency Intensive Care Unit, Changshu Hospital Affiliated to Soochow University, Changshu, China; ^2^Department of Neurosurgery, Changshu Hospital Affiliated to Soochow University, Changshu, China; ^3^School of Computer Science, Nanjing University of Posts and Telecommunications, Nanjing, China; ^4^Department of Neurosurgery, Changshu No.2 People's Hospital, The Affiliated Changshu Hospital of Nantong University, Changshu, China; ^5^Intelligent Medical Technology Research Center, Changshu Hospital Affiliated to Soochow University, Changshu, China; ^6^Department of Neurosurgery, Ji'an Central People's Hospital, Ji'an, China

**Keywords:** spontaneous intracerebral hemorrhage, machine learning, prognostic prediction, XGBoost, SHAP

## Abstract

**Background:**

Spontaneous intracerebral hemorrhage (SICH) is the second most common cause of cerebrovascular disease after ischemic stroke, with high mortality and disability rates, imposing a significant economic burden on families and society. This retrospective study aimed to develop and evaluate an interpretable machine learning model to predict functional outcomes 3 months after SICH.

**Methods:**

A retrospective analysis was conducted on clinical data from 380 patients with SICH who were hospitalized at three different centers between June 2020 and June 2023. Seventy percent of the samples were randomly selected as the training set, while the remaining 30% were used as the validation set. Univariate analysis, Least Absolute Shrinkage and Selection Operator (LASSO) regression, and Pearson correlation analysis were used to screen clinical variables. The selected variables were then incorporated into five machine learning models: complementary naive bayes (CNB), support vector machine (SVM), gaussian naive bayes (GNB), multilayer perceptron (MLP), and extreme gradient boosting (XGB), to assess their performance. Additionally, the area under the curve (AUC) values were evaluated to compare the performance of each algorithmic model, and global and individual interpretive analyses were conducted using importance ranking and Shapley additive explanations (SHAP).

**Results:**

Among the 380 patients, 95 ultimately had poor prognostic outcomes. In the validation set, the AUC values for CNB, SVM, GNB, MLP, and XGB models were 0.899 (0.816–0.979), 0.916 (0.847–0.982), 0.730 (0.602–0.857), 0.913 (0.834–0.986), and 0.969 (0.937–0.998), respectively. Therefore, the XGB model performed the best among the five algorithms. SHAP analysis revealed that the GCS score, hematoma volume, blood pressure changes, platelets, age, bleeding location, and blood glucose levels were the most important variables for poor prognosis.

**Conclusion:**

The XGB model developed in this study can effectively predict the risk of poor prognosis in patients with SICH, helping clinicians make personalized and rational clinical decisions. Prognostic risk in patients with SICH is closely associated with GCS score, hematoma volume, blood pressure changes, platelets, age, bleeding location, and blood glucose levels.

## 1 Introduction

Spontaneous intracerebral hemorrhage (SICH) refers to the non-traumatic rupture of blood vessels within the brain, leading to the accumulation of blood within the brain parenchyma (1). ICH is the second most common cause of cerebrovascular disease after ischemic stroke, with the incidence of hemorrhagic stroke rising in recent years, especially in developing countries, at approximately 15–30 cases per 100,000 people (2, 3). Due to its high mortality and morbidity rates, only about 20% of patients are regain functional independence 6 months post-stroke, imposing a significant burden on individuals, families, and society (4). Despite advancements in diagnostic and therapeutic technologies in recent years, predicting the prognosis of patients with SICH remains a clinical challenge.

Previous studies have attempted to identify factors affecting the prognosis of ICH. Hemphill and colleagues proposed the ICH Score, a predictive tool that predicts mortality (5). However, the ICH Score only includes factors such as the Glasgow Coma Scale (GCS), ICH volume, age, location, and ventricular extension of the hematoma. Recent studies have found that other factors, such as laboratory indicators and medication usage, are also related to the outcome of ICH (6, 7). Furthermore, the predictive value of different factors has been inconsistent across studies, partly because these studies often rely on traditional statistical methods, which have limitations in dealing with complex clinical data.

With the increasing application of Artificial Intelligence (AI) in the field of medicine, especially the development of Machine Learning (ML) technologies, new possibilities for predicting the prognosis of ICH have emerged. The advantage of ML models is their ability to process large amounts of data and identify complex patterns, which is crucial for understanding the multifactorial nature of ICH and improving prediction accuracy (8). In recent years, ML has been increasingly applied in predicting outcomes and prognoses in cerebrovascular diseases such as subarachnoid hemorrhage (9) and acute ischemic stroke (10). However, many ML models often lack interpretability in their predictions, limiting their application in clinical practice (11). This study aims to assess the effectiveness of ML models in predicting the functional prognosis of ICH patients. We utilize explainable ML models to improve the precision and transparency of patient prognosis predictions. We focus on how the model interprets individualized patient risks in terms of clinical significance and explore its potential value in guiding treatment decisions and improving patient management.

## 2 Materials and methods

### 2.1 Study population and data collection

This retrospective study recruited patients with SICH who were hospitalized between January 2021 and January 2023 at three hospitals: Soochow University Affiliated Changshu Hospital, Nantong University Affiliated Changshu Hospital, and Changshu City Traditional Chinese Medicine Hospital. Each patient was monitored for at least 3 months after the onset of the disease. All follow-ups and prognostic evaluations for patients included in the study from the three hospitals in the region were conducted according to the same standards by the regional Health Commission. Based on the follow-up results after 3 months, patients were divided into groups with good functional prognosis and poor functional prognosis. A modified Rankin Scale (mRS) score of 3–6 was defined as a poor prognostic outcome, while an mRS score of 0–2 was defined as a good prognostic outcome (12). Because this study had a retrospective design, there was no security-related risk. The present study was approved by the Ethics Committee of the Soochow University Affiliated Changshu Hospital and confirmed to the declaration of Helsinki.

**Figure 1:**
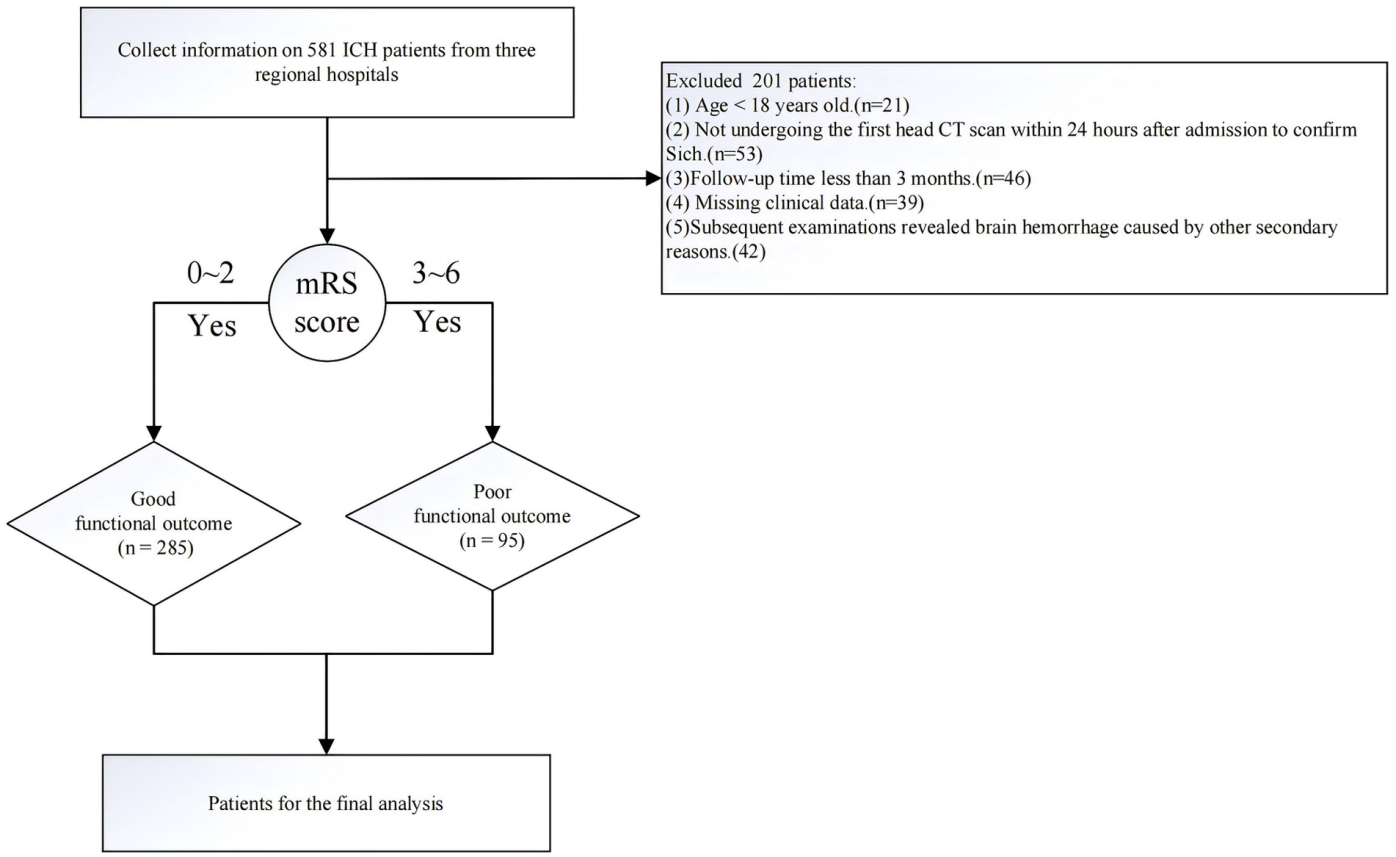
Flowchart of the data filtering process.

The inclusion criteria for the study were as follows: The inclusion criteria for this study were as follows: (1) Intracerebral hemorrhage confirmed by head CT scan. (2) Patient age ≥ 18 years. (3) Complete medical records (including baseline data, laboratory tests, imaging data, treatment records, and outcome data). The exclusion criteria were: (1) Intracerebral hemorrhage caused by secondary factors, such as traumatic brain injury, brain tumors leading to stroke, coexisting cerebrovascular diseases (e.g., intracranial aneurysms or arteriovenous malformations), or hemorrhagic transformation of cerebral infarction. (2) Death within 7 days of hospitalization or discharge directly to home from the hospital. (3) Severe coagulation disorders or coagulopathy caused by malignancies or liver dysfunction. (4) Patients lacking sufficient follow-up data. The flowchart of patient inclusion in this study is shown in Figure 1.

### 2.2 Inclusion of observed variables

Clinical data were collected from patients based on the clinical experience of the three centers, literature reports, and the electronic medical record system. The following data were primarily collected: (1) Demographic data (gender, age); (2) Past medical history (history of hypertension, use of antithrombotic drugs, diabetes, heart disease, history of cerebral infarction, previous cerebral hemorrhage, trauma history, history of uremia, chronic liver disease history, smoking history, alcohol consumption history, etc.); (3) Baseline vital signs at admission: systolic blood pressure (SBP), diastolic blood pressure (DBP), Glasgow Coma Scale (GCS) score; (4) Baseline disease characteristics (time from onset to emergency room, blood pressure reduction treatment in the emergency room, location of hemorrhage, hematoma volume); (5) Baseline laboratory test data (international normalized ratio (INR), prothrombin time (PT), activated partial thromboplastin time (APTT), thrombin time (TT), fibrinogen (Fbg), D-dimer, hemoglobin (HGB), platelets (PLT), blood glucose). (5) Treatment-related indicators: SBP change from emergency room to hospital admission, DBP change from emergency room to hospital admission, treatment methods (medication and different surgical methods). Location of the hematoma, intraventricular hemorrhage, and the initial hematoma volume were evaluated by CT scan independently by two experienced doctors. The hematoma volume was measured using the ABC/2 method, in which A is the greatest diameter on the largest hemorrhage slice, B is the diameter perpendicular to A, and C is the approximate number of axial slices with hemorrhage multiplied by the slice thickness. Inpatient treatment includes conservative treatment or surgical intervention. Generally, surgical treatment is recommended for patients with supratentorial hematomas ≥30 mL or infratentorial hematomas ≥10 mL. Neurosurgeons choose different surgical methods based on the patient’s condition, including microscopic hematoma removal surgery, neuroendoscopic hematoma removal surgery, and neuroguided puncture and drainage of hematoma.

### 2.3 Selection of machine learning models

Before constructing ML models, the original clinical data were normalized. Normalization can improve the speed of gradient descent to find the optimal solution, and the algorithm for Euclidean distance can effectively improve the accuracy. In this study, the min–max normalization method was used to normalize the characteristic values of clinical data to the range of (0, 1). Categorical variables were one-hot encoded, converting each category into a binary variable that is mutually exclusive. These one-hot encoded variables were then added to the model’s feature matrix as input features. This encoding method not only retains the original classification information but also allows for effective input into the ML model.

The study population was divided into a training set and a test set at a 7:3 ratio using a simple random sampling method. The training set was used to build models to predict the functional outcomes of ICH patients at 3 months, and the test set was used for internal validation. All variables were included in the Least Absolute Shrinkage and Selection Operator (LASSO) regression for variable selection (13). Then, the selected variables were incorporated into different machine-learning algorithms to construct predictive models. LASSO regression is a type of linear regression model that uses a mathematical approach (adding L1 regularization) to shrink the coefficients of some less important features to zero, thereby automatically selecting the features that contribute the most to the predictive outcome. This method not only simplifies the model and makes it easier to interpret but also prevents the model from becoming overly complex, reducing the risk of overfitting.

Five ML algorithms were applied for data modeling: Complementary Naive Bayes (CNB), Support Vector Machine (SVM), Gaussian Naive Bayes (GNB), Multilayer Perceptron (MLP), and Extreme Gradient Boosting (XGB). The CNB model is suitable for high-dimensional data and performs well when handling sparse data. However, CNB is not suitable for strongly correlated features, which limits its predictive performance on complex problems. The SVM model can handle high-dimensional features and is suitable for small sample sizes. However, it is less efficient for large datasets and multi-class problems, and it is sensitive to missing data. The GNB model is computationally efficient and suitable for simple problems. However, it has limited ability to model complex relationships. The MLP model can handle complex nonlinear relationships and high-dimensional data, making it suitable for large datasets and multi-task learning. However, it requires long training times, large amounts of data, and significant computational resources, and is prone to overfitting when sample sizes are small. The XGB model is an ensemble learning algorithm capable of handling complex nonlinear relationships and multiple feature interactions. It requires numerous experiments and sufficient training time. To ensure that the training samples selected for multiple model training are consistent, we used a resampling training/validation mechanism to extend each model’s performance across multiple training processes. The criteria for hyperparameters were primarily based on model performance evaluation metrics, including accuracy, precision, recall, F1 score, and the area under the ROC curve (AUC-ROC). For the CNB algorithm, the “alpha” smoothing parameter was set to 1; in the XGB algorithm, the maximum tree depth was set to 4 nodes, the learning rate to 3, and the minimum split weight to 6; for the SVM algorithm, the kernel type was chosen as “RBF” and the regularization parameter was set to 1.0. For the MLP algorithm, there were 10 iterations, with 30 neurons per layer in the hidden layers and the activation function type was logistic. In the GNB algorithm, “var_smoothing” was set to 1e-7. We aimed to improve model predictive performance by finding the optimal combination of hyperparameters.

After selecting and evaluating the ML models, this study further utilizes SHAP analysis to interpret the model’s prediction results. SHAP values, based on the Shapley value from cooperative game theory, provide a method for explaining the output of ML models by quantifying the contribution of each feature to the prediction result. All the included features collectively predict the outcome, and SHAP values assess each feature’s “marginal contribution,” i.e., the effect of adding the feature alone or in combination with others to the prediction. SHAP values indicate which features have a positive or negative impact on a specific prediction and the magnitude of their influence. This helps understand the model’s decision-making process and enhances trust in ML models in clinical tasks. Additionally, SHAP values can be used to explain the prediction results for individual patients and identify the key factors influencing prognosis. By providing a detailed analysis of the key factors influencing patients’ functional prognosis, SHAP offers valuable insights for clinical decision-making, thereby enhancing the clinical applicability of the model.

### 2.4 Statistical analysis

In this study, statistical analysis was conducted on all variables across the two groups. R software (version 4.02) was used for data processing and statistical analysis. Categorical variables were presented as counts and percentages and compared using Fisher’s exact test or chi-square test. For continuous variables, the Shapiro–Wilk test was first used to determine if the variables followed a normal distribution, followed by the independent samples t-test (for normally distributed data) for data comparison, represented as mean ± standard deviation. The Mann–Whitney U test was used for comparing non-normally distributed data, represented by median (first and third quartiles). A *p*-value of <0.05 was considered statistically significant.

## 3 Results

### 3.1 Baseline patient characteristics

This study included a total of 380 patients, with baseline characteristics of the good prognosis group and the poor prognosis group after cerebral hemorrhage shown in Table 1. The median age of the patients in this study was 65 (52.00, 75.00) years. In terms of gender distribution, there were 264 males (69.47%) and 116 females (30.53%). Detailed baseline characteristics of the patient’s medical history, vital signs, relevant laboratory indicators, and treatment-related indicators are available in Table 1. Comparison of baseline characteristics between the two groups showed statistical differences in age, history of diabetes, history of uremia, time from onset to arrival at the emergency room, SBP measured in the emergency room, DBP measured in the emergency room, treatment with intravenous blood pressure reduction in the emergency room, location of hemorrhage, volume of hematoma, change in SBP, change in DBP, GCS, D-dimer, blood glucose, treatment methods, and perioperative blood pressure control (*p* < 0.05).

**Table 1:** Clinical characteristics of the patient cohort included in this study.

Variables	Total(*n* = 380)	Unfavorable outcome(*n* = 95)	Favorable outcome(*n* = 285)	*p* value
Age, median (Q1,Q3) (years)	65(52.00,75.00)	60.000(50.00,74.00)	66.000(53.00,75.00)	0.048
Sex, n (%)				0.797
Male	264(69.47)	65(68.42)	199(69.83)	
Female	116(30.53)	30(31.58)	86(30.18)	
Hypertension, n (%)				0.203
No	120(31.58)	35(36.84)	85(29.82)	
Yes	260(68.42)	60(63.16)	200(70.18)	
Taking antihypertensive drugs, n (%)				0.765
No	163(42.89)	42(44.21)	121(42.46)	
Yes	217(57.11)	53(55.79)	164(57.54)	
Taking antithromboticdrugs, n (%)				0.159
No	338(88.95)	85(89.47)	253(88.77)	
Warfarin	4(1.05)	2(2.11)	2(0.71)	
Aspirin	27(7.10)	3(3.16)	24(8.42)	
Clopidogrel	9(2.37)	4(4.21)	5(1.75)	
Others	2(0.53)	1(1.05)	1(0.35)	
Diabetes, n (%)				<0.001
No	332(87.37)	88(92.63)	244(85.61)	
Yes	48(12.63)	7(7.37)	41(14.39)	
Heart disease, n(%)				0.596
No	369(97.11)	93(97.89)	276(96.84)	
Yes	11(2.89)	2(2.11)	9(3.16)	
Cerebral infarction, n (%)				0.102
No	334(87.89)	88(92.63)	246(86.32)	
Yes	46(12.11)	7(7.37)	39(13.68)	
Cerebral hemorrhage, n (%)				0.299
No	353(92.89)	86(90.53)	267(93.68)	
Yes	27(7.11)	9(9.47)	18(6.32)	
Craniocerebral trauma, n (%)				0.795
No	375(98.68)	94(98.95)	281(98.60)	
Yes	5(1.32)	1(1.05)	4(1.40)	
Uremia, n (%)				<0.001
No	362(95.26)	80(84.21)	282(98.95)	
Yes	18(4.74)	15(15.79)	3(1.05)	
History of malignant tumors, n (%)				0.753
No	366(96.32)	92(96.84)	274(96.14)	
Yes	14(3.68)	3(3.16)	11(3.86)	
History of epilepsy, n (%)				0.509
No	373(98.16)	94(98.95)	279(97.89)	
Yes	7(1.84)	1(1.05)	6(2.11)	
Chronic Liver Disease, n (%)				1.0
No	376(98.95)	94(98.95)	282(98.95)	
Yes	4(1.05)	1(1.05)	3(1.05)	
History of drinking, n (%)				0.21
No	301(79.21)	71(74.74)	230(80.70)	
Yes	79(20.79)	24(25.26)	55(19.30)	
History of smoking, n (%)				0.261
No	292(76.84)	69(72.63)	223(78.25)	
Yes	88(23.16)	26(27.37)	62(21.75)	
Time from onset to the emergency room, median (Q1,Q3; h)	2.00(1.12,5.17)	1.33(0.95,2.00)	2.33(1.23,8.28)	<0.001
Emergency room SBP, Mean ± SD (mmHg)	176.74 ± 29.34	193.70 ± 30.10	171.09 ± 26.80	<0.001
Emergency room DBP, median (Q1,Q3; mmHg)	96.000(85.00,107.00)	104.00(90.00,120.00)	94.00(83.00,104.00)	<0.001
Emergency Room Venous Hypotension Treatment, n (%)				0.031
No	177(46.58)	33(34.74)	144(50.53)	
Urapidil	173(45.53)	51(53.68)	122(42.81)	
Nicardipine	22(5.79)	7(7.37)	15(5.26)	
Sodium Nitroprusside	8(2.11)	4(4.21)	4(1.40)	
Cerebral hemorrhage site, n (%)				0.005
Basal ganglia	233(61.32)	62(65.26)	171(60.00)	
Lobe	79(20.79)	11(11.58)	68(23.86)	
Cerebellum	16(4.21)	2(2.11)	14(4.91)	
Brainstem	14(3.68)	3(3.16)	11(3.86)	
Others	38(10.00)	17(17.90)	21(7.37)	
Intraventricular hemorrhage, n (%)				0.364
No	334(87.90)	81(85.26)	253(88.77)	
Yes	46(12.11)	14(14.74)	32(11.23)	
Hematoma volume, median (Q1,Q3; mL)	10.99(5.25,26.92)	35.00(18.60,75.02)	7.30(4.37,16.19)	<0.001
Hospitalized SBP, median (Q1,Q3; mmHg)	154.00(141.00,172.00)	160.00(142.00,175.00)	154.00(141.00,170.00)	0.236
Hospitalized DBP, median (Q1,Q3; mmHg)	90.00(80.00,98.00)	92.00(81.00,100.00)	89.00(80.00,98.00)	0.208
SBP change, median (Q1,Q3; mmHg)	20.00(12.00,35.00)	40.00(27.00,54.00)	16.00(10.00,27.00)	<0.001
DBP change, median (Q1,Q3; mmHg)	10.00(5.00,19.00)	19.00(11.00,28.00)	9.00(5.00,15.00)	<0.001
GCS, median (Q1,Q3)	12.00(10.00,15.00)	7.00(5.00,8.00)	14.00(13.00,15.00)	<0.001
INR, median (Q1,Q3)	1.01(0.95,1.06)	1.00(0.95,1.06)	1.01(0.96,1.06)	0.555
PT, median (Q1,Q3)(s)	12.80(11.70,13.40)	12.80(12.00,13.40)	12.70(11.60,13.40)	0.284
APTT, median (Q1,Q3)(s)	31.70(28.20,35.10)	31.20(28.60,35.20)	31.80(28.20,34.90)	0.998
TT, median (Q1,Q3)(s)	17.300(16.10,18.50)	17.600(16.50,19.00)	17.300(15.90,18.40)	0.053
Fbg, median (Q1,Q3; g/L)	3.04(2.59,3.69)	3.06(2.58,3.77)	3.04(2.61,3.68)	0.723
D-Dimer, median (Q1,Q3; mg/L)	0.35(0.17,0.78)	0.45(0.26,0.93)	0.30(0.15,0.70)	0.003
HGB, median (Q1,Q3; g/L)	138.00(125.00,151.00)	139.00(124.00,151.00)	137.00(125.00,150.00)	0.690
PLT, median (Q1,Q3; 10^9^/L)	174.00(133.00,217.00)	172.000(117.00,218.00)	174.00(136.00,217.00)	0.424
Blood glucose, median (Q1,Q3; mmol/L)	7.69(6.19,9.57)	8.360(7.42,11.02)	7.23(6.01,9.09)	<0.001
Treatment methods, n (%)				<0.001
Conservative drug therapy	308(81.05)	53(55.79)	255(89.47)	
Microscopic hematoma removal surgery	35(9.21)	22(23.16)	13(4.56)	
Neuroendoscopic hematoma removal surgery	19(5.00)	11(11.58)	8(2.81)	
Neuroguided puncture and drainage of hematoma	18(4.74)	9(9.47)	9(3.16)	
Perioperative blood pressure control, n (%)				<0.001
No	294(77.37)	55(57.90)	239(83.86)	
Yes	86(22.63)	40(42.10)	46(16.14)	

SBP, systolic blood pressure; DBP, diastolic blood pressure; GCS, Glasgow Coma Scale; INR, International Normalized Ratio; PT, Prothrombin Time; APTT, Activated Partial Thromboplastin Time; TT, Thrombin Time; Fbg, Fibrinogen; HGB, Hemoglobin; PLT, Platelets.

### 3.2 Variable filtering

**Figure 2:**
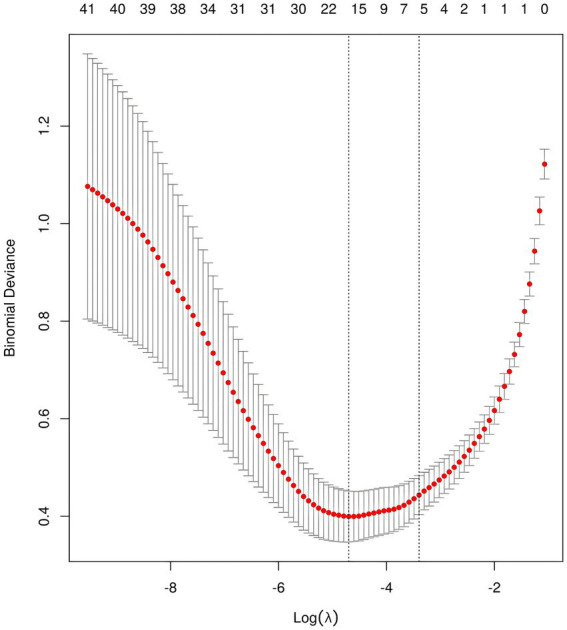
LASSO feature selection for model construction.

**Figure 3:**
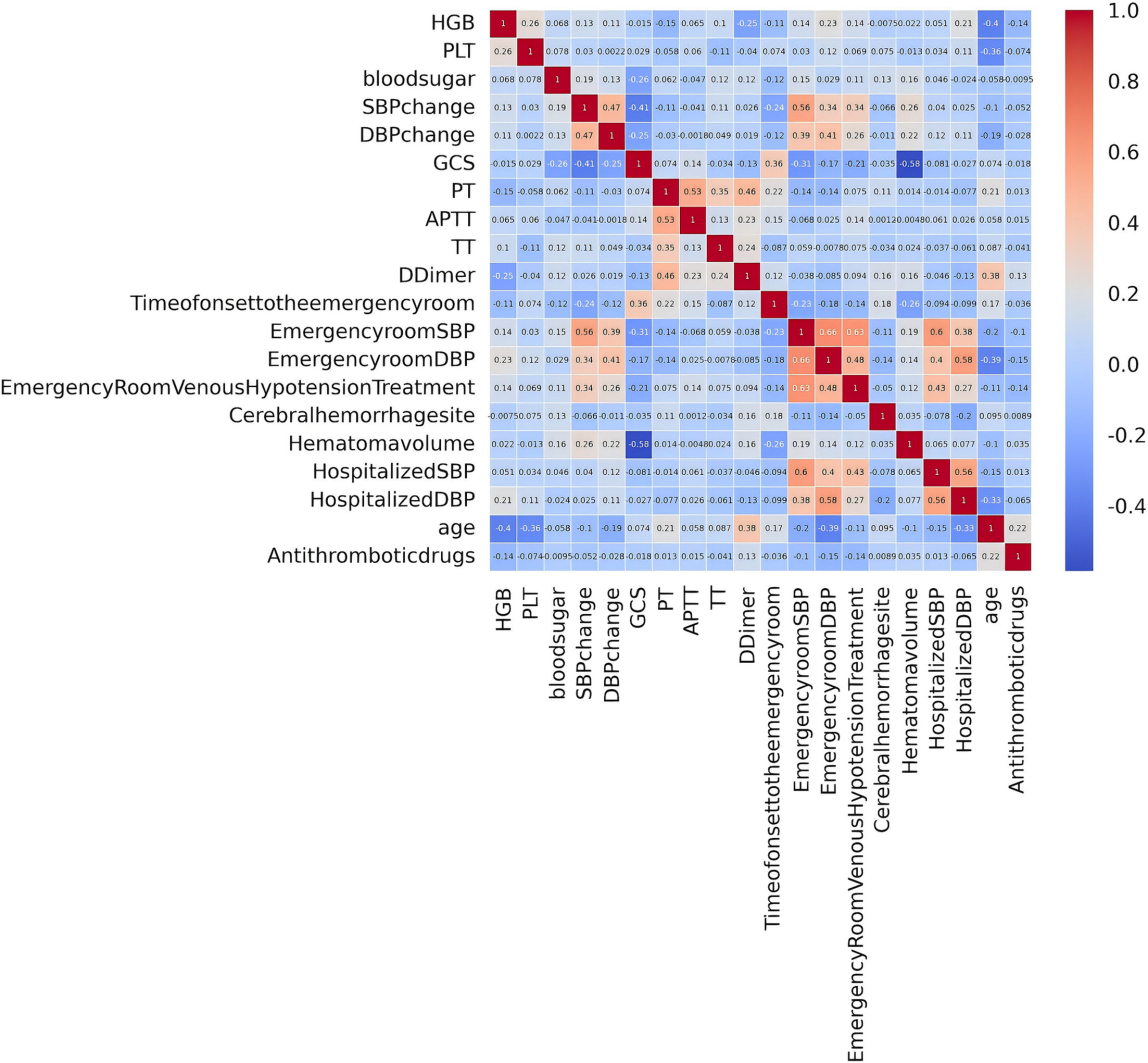
Spearman correlation analysis among variables.

After performing LASSO regression analysis on the training set, the features were reduced to 20 potential predictive variables (Figure 2). We further constructed a heatmap to display Spearman correlation coefficients, visualizing the correlations with the differential variables (Figure 3). A Spearman correlation heatmap is a visualization tool that helps quickly identify and display monotonic relationships between different features through correlation coefficients, not just linear relationships. The deeper the color, the stronger the correlation. By identifying highly correlated feature pairs, it can help filter out and remove redundant features. These features specifically include GCS, age, hematoma volume, use of antithrombotic and anticoagulant drugs, SBP at the emergency room, DBP at the emergency room, SBP at admission, DBP at admission, change in SBP, change in DBP, treatment with intravenous blood pressure reduction in the emergency room, location of hemorrhage, time from onset to arrival at the emergency room, APTT, PT, TT, PLT, HGB, and D-dimer. In our study, the threshold for feature correlations was set below 0.7, indicating that there are no highly correlated features and the feature variables are not redundant. Based on these findings, we decided to incorporate the results of the LASSO regression analysis into our machine-learning algorithms for further analysis.

### 3.3 Comparison of the predictive performance of all models

**Figure 4:**
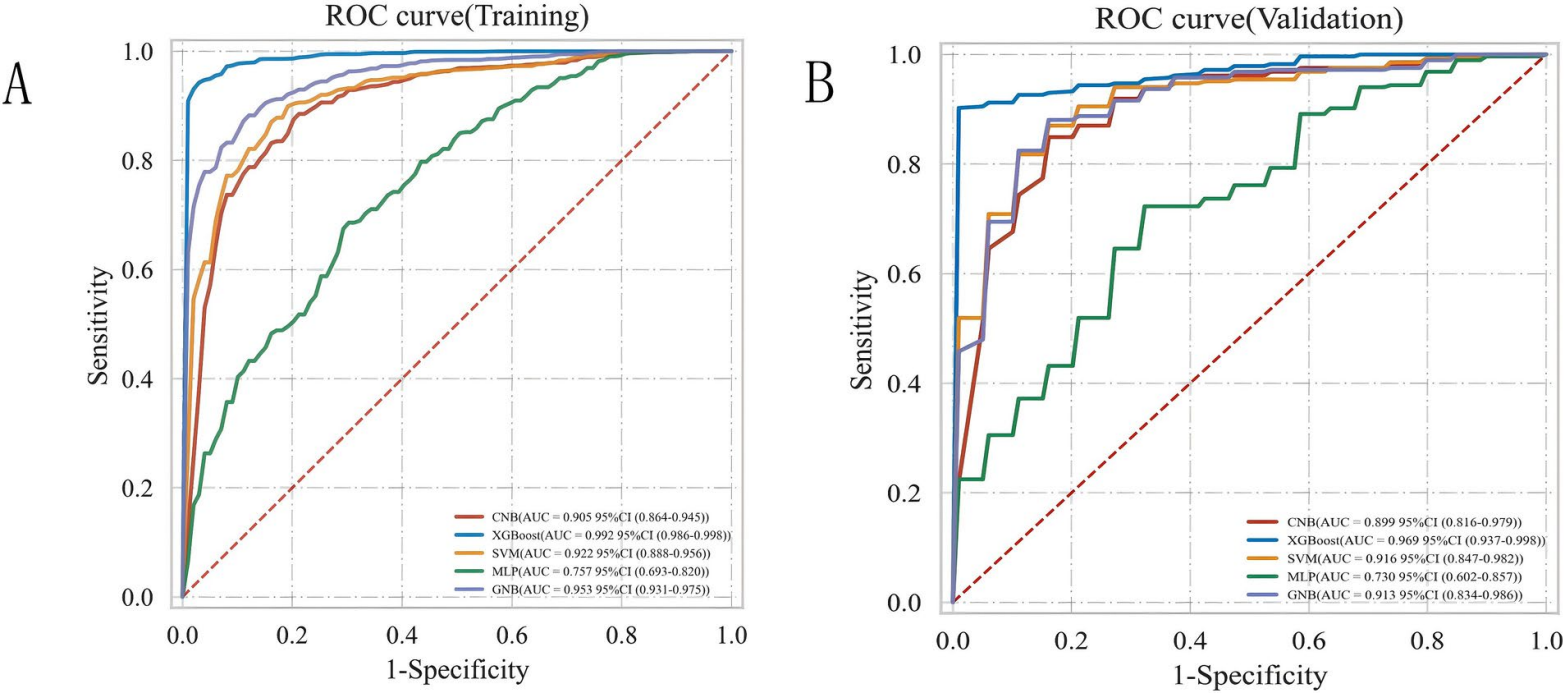
ROC curve analysis of five ML algorithms for training set **(A)** and test set **(B)**.

**Figure 5:**
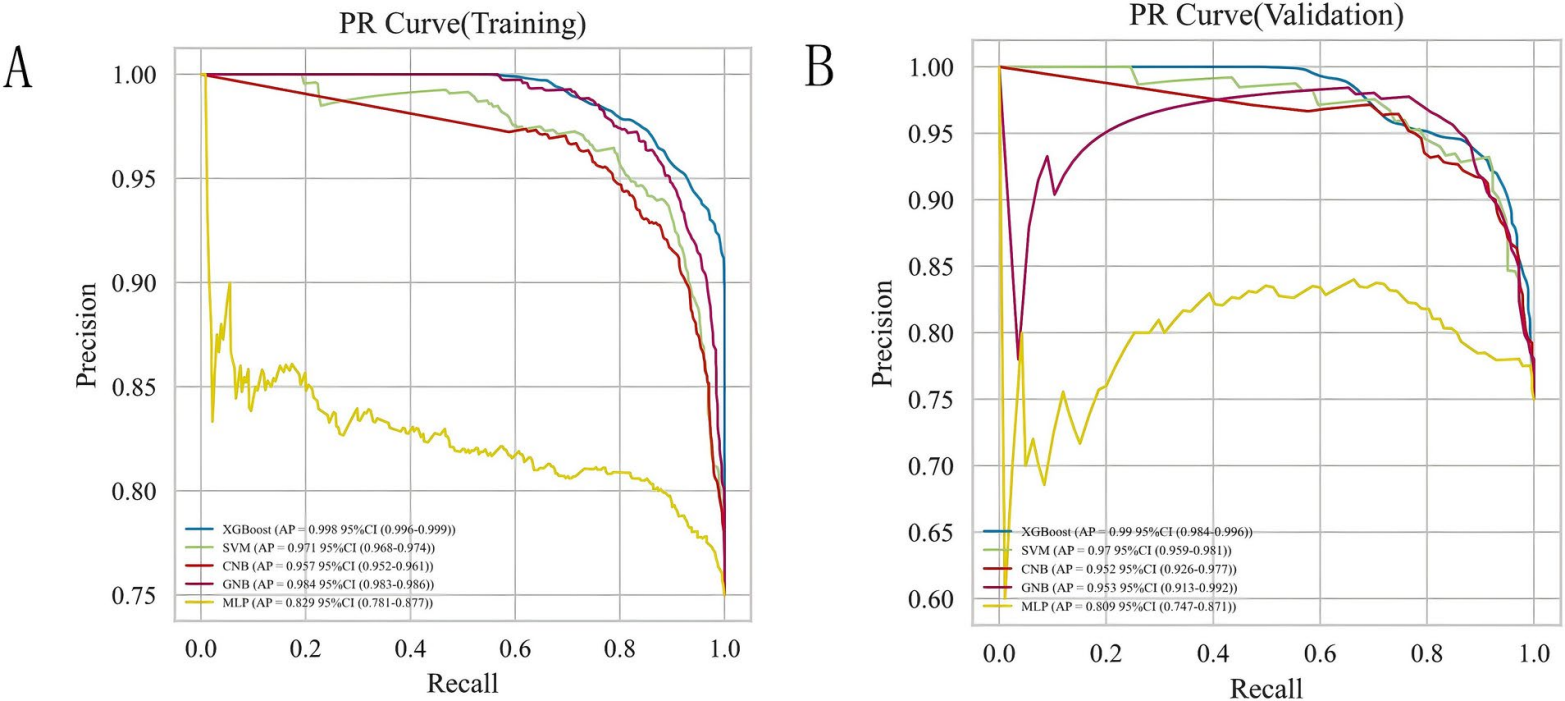
Analysis of PR curves of five ML algorithms for training set **(A)** and test set **(B)**.

To achieve the best predictive model, this study employed five different ML algorithms: CNB, XGB, SVM, MLP, and GNB. The training set was used to create and train the models. All ML models were tested in the test set, and their accuracy, precision, sensitivity, specificity, and F1 score were compared. The XGB model demonstrated the highest accuracy, precision, sensitivity, specificity, and F1 score (respectively, 0.9, 0.985, 0.902, 1.0, and 0.928; Table 2). From Figures 4A,B, it is evident that the XGB algorithm has a higher AUC value in both the training set (AUC = 0.992; 95% CI, 0.986–0.998) and the test set (AUC = 0.969; 95% CI, 0.937–0.998) compared to the other four algorithms. Additionally, the area under the PR curve for the XGB algorithm was also the highest in both the training set (0.998; 95% CI, 0.996–0.999) and the test set (0.99; 95% CI, 0.984–0.996; Figures 5A,B).

**Table 2:** Comparison of the predictive performance of five machine learning algorithms in the validation set.

Different algorithms	accuracy (95%CI)	precision (95%CI)	Sensitivity (95%CI)	Specificity (95%CI)	F1-score (95%CI)	AUC (95%CI)
CNB	0.826(0.793–0.859)	0.927(0.907–0.946)	0.832(0.743–0.920)	0.895(0.849–0.941)	0.875(0.818–0.932)	0.899 (0.816–0.979)
XGB	0.9(0.883–0.917)	0.958(0.924–0.991)	0.902(0.866–0.937)	1.0(1.000–1.000)	0.928(0.913–0.943)	0.969 (0.937–0.998)
SVM	0.834(0.762–0.907)	0.943(0.921–0.965)	0.853(0.762–0.943)	0.884(0.846–0.923)	0.893(0.836–0.950)	0.916 (0.847–0.982)
MLP	0.724(0.664–0.783)	0.841(0.776–0.906)	0.73(0.579–0.880)	0.737(0.555–0.918)	0.775(0.672–0.878)	0.730 (0.602–0.857)
GNB	0.855(0.810–0.901)	0.952(0.908–0.995)	0.842(0.799–0.886)	0.937(0.886–0.987)	0.893(0.858–0.928)	0.913 (0.834–0.986)

CNB, Complement Naive Bayes; XGB, extreme gradient boost; SVM, support vector machine; MLP, Multilayer Perceptron; GNB, Gaussian Naive Bayes.

As shown in Table 3, the performance of the ICH score, calculated using the previously described method (5), was compared to that of the developed ML-based model. Additionally, we tested the performance of a traditional logistic regression model. The results showed that compared to the ICH score (AUC = 0.874; 95% CI, 0.841–0.908) and the logistic regression model (AUC = 0.849; 95% CI, 0.725–0.969), the XGB model had a higher AUC value (0.969), demonstrating superior predictive performance over both the traditional logistic regression model and the ICH score.

**Table 3:** Predictive performance of functional outcome after spontaneous intracerebral hemorrhage.

Algorithm	Functional outcome	
	AUC(Mean)	AUC (95%CI)
ICH score	0.874	(0.841–0.908)
LR	0.849	(0.725–0.969)
CNB	0.899	(0.816–0.979)
XGB	0.969	(0.937–0.998)
SVM	0.916	(0.847–0.982)
MLP	0.730	(0.602–0.857)
GNB	0.913	(0.834–0.986)

LR, Logistic Regression; CNB, Complement Naive Bayes; XGB, extreme gradient boost; SVM, support vector machine; MLP, Multilayer Perceptron; GNB, Gaussian Naive Bayes.

**Figure 6:**
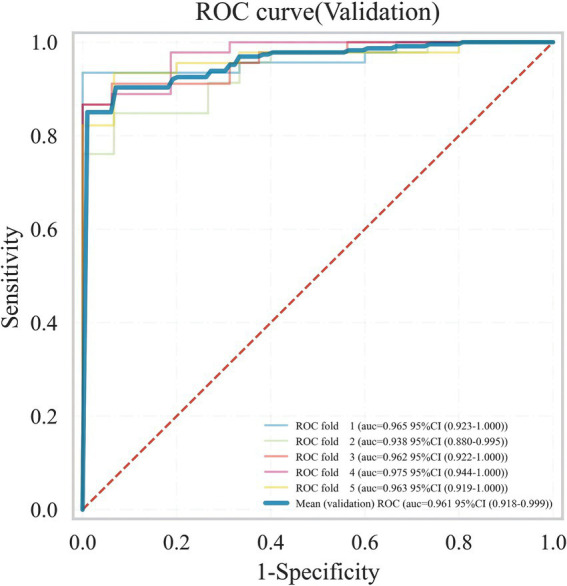
ROC plot of XGB ML model 5-fold cross-validation results.

**Figure 7:**
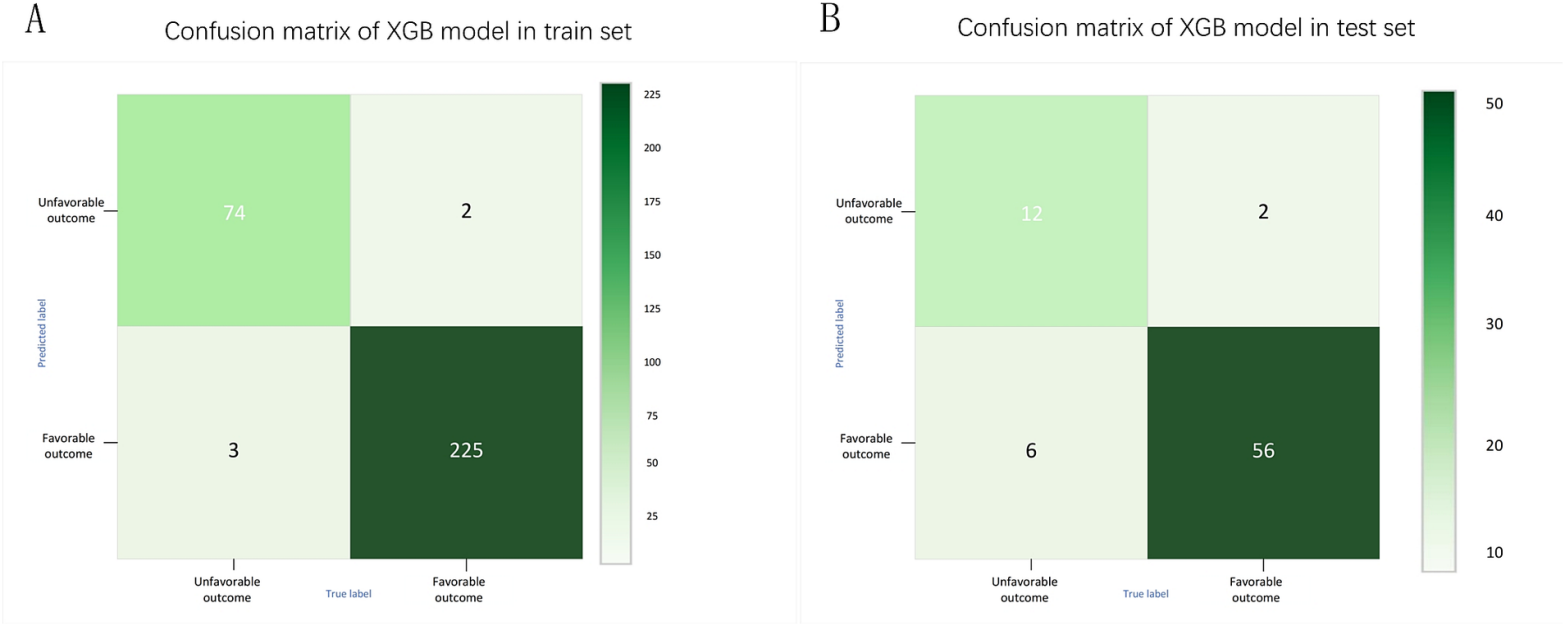
Confusion matrix of XGB model in training set **(A)** and test set **(B)**.

Meanwhile, considering that in real clinical settings, the prognosis of SICH patients is influenced by various factors, including medical history, treatment methods, and imaging data, XGB effectively models the complex nonlinear relationships among these factors. By integrating multiple decision trees, XGB enhances the predictive accuracy of the model and demonstrates greater robustness in addressing complex clinical tasks such as predicting the prognosis of SICH patients. Therefore, the XGB algorithm was chosen to select predictive factors for the prognosis of SICH patients. To understand the performance of the XGB algorithm model, 5-fold cross-validation was used as a resampling method for internal validation within the training set data. In each iteration, four folds were used as the training subset, with the remaining one used for parameter tuning. As shown in Figure 6, the validation results indicated that the XGB algorithm has good predictive power (AUC = 0.961, 95% CI, 0.918–0.999). Moreover, Figures 7A,B displays the confusion matrix of the optimal classifier in XGB.

### 3.4 Explainable analysis of overall features

**Figure 8:**
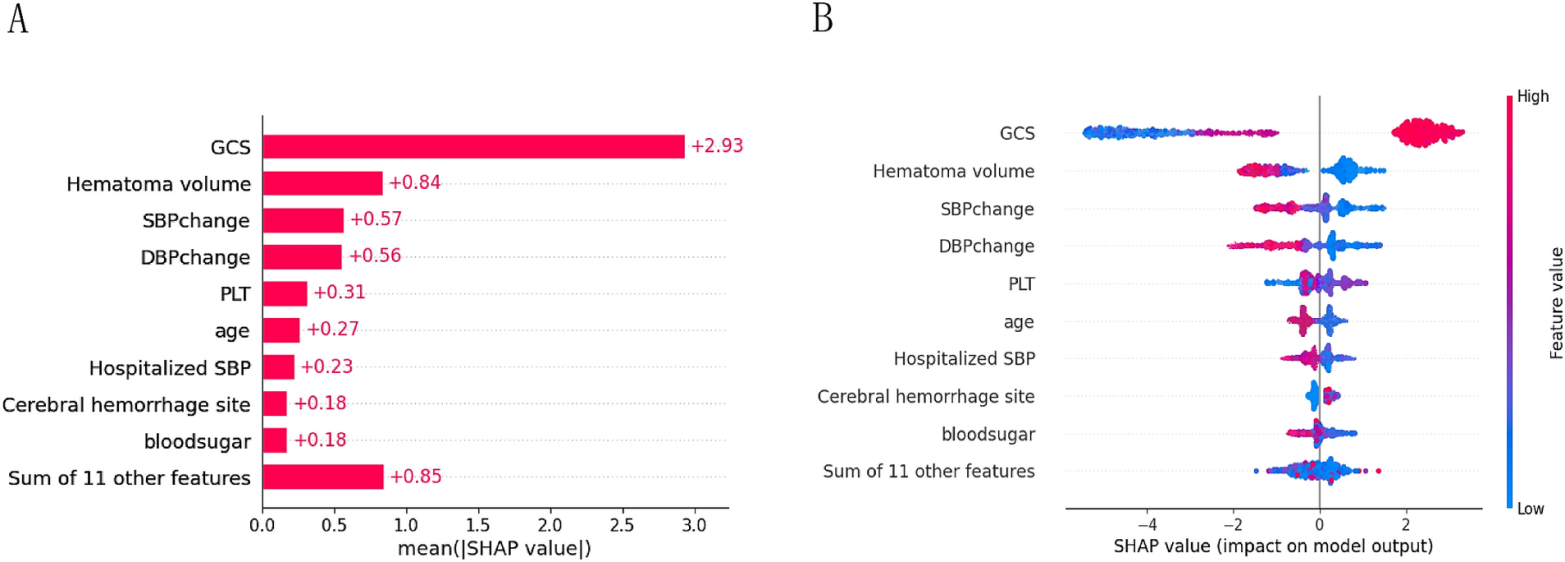
Importance ranking plot **(A)** and scatter plot of variables for SHAP analysis **(B)**.

**Figure 9:**
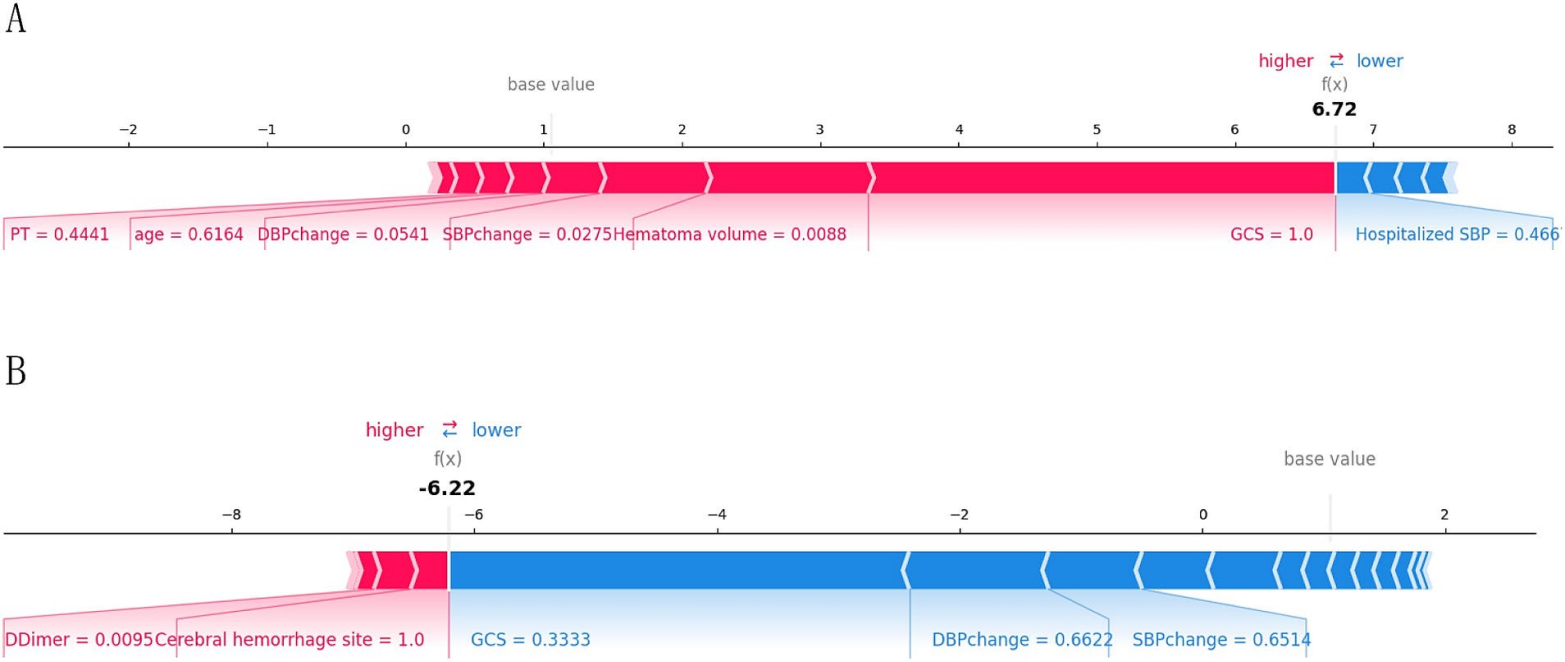
Interpretation of the SHAP model for the prediction of two cases. **(A)** Contribution of different features to the correct prediction of poor prognosis in ICH patients. **(B)** Contribution of different features to the correct prediction of good prognosis in ICH patients.

To provide a more intuitive and clear explanation of the XGB model, we used SHAP to rank the importance of features. Figure 8A depicts the ranking of variables in the XGB model according to their importance. The feature importance plot (Figure 8B) shows how the main feature variables in the dataset influence the final output of the model. On the left y-axis, features are ranked according to their importance; on the right y-axis, red dots represent higher feature values, while blue dots represent lower feature values. The x-axis represents SHAP values, showing the contribution of features to the overall output. Results indicate that lower GCS scores, larger hematoma volumes, greater blood pressure changes, and older age have lower SHAP values, suggesting a higher likelihood of poor prognosis for the patient. Figures 9A,B demonstrate that SHAP can also be used to analyze model interpretations for individual patients. Arrows in the figure show the impact of each factor on the prediction. Features increasing the risk of poor prognosis are represented in red, while features decreasing the risk are represented in blue. The length of each feature’s bar reflects the extent of its contribution to the predictive outcome.

## 4 Discussion

SICH is a common and severe neurosurgical disease characterized by high morbidity and mortality rates. With the development of Chinese society and the increasing degree of population aging, cerebrovascular diseases are becoming a significant burden on society (14). In this study, we included data from three regional medical institutions and successfully constructed a predictive model for the 3-month prognosis of SICH patients using the XGB algorithm. Additionally, we applied five mainstream ML models, including CNB, XGB, SVM, MLP, and GNB, to compare the predictive performance differences between models, ultimately utilizing SHAP analysis to visualize and analyze model predictions.

In recent years, many studies have applied ML algorithms to predict the functional prognosis, related complications, and treatment responses in patients with cerebrovascular diseases. These studies not only help us understand the wide application of ML in the field of neurology but also provide insights into the clinical practice of predicting cerebrovascular diseases. XGB, an ensemble learning method based on decision tree algorithms, can reveal complex patterns and relationships in data. Given that medical data often contain a large number of nonlinear relationships and high-dimensional features, XGB becomes an efficient and flexible solution for handling big medical data (15). Previous studies include one by Li et al., who developed an XGB-based ML model to predict the risk of hemorrhagic transformation and death during thrombolysis for acute cerebral infarction, achieving AUC values of 0.95 and 0.85, respectively (16). Our team previously constructed an ML model to predict the risk of cerebral hemorrhage in patients with long-term hemodialysis uremia, achieving an AUC value of 0.969 in the validation set, along with a visual interpretative analysis of risk factors (17). Gu’s team applied ML algorithms to identify efficient predictors of early mortality in non-traumatic subarachnoid hemorrhage patients in the ICU, with an external validation AUC value of 0.913 (9). The results of the current study show that the XGB model performs best in the test set, with the highest accuracy, sensitivity, specificity, and F1 score (0.9, 0.985, 0.902, 1.0, and 0.928, respectively), and an ROC-AUC value of 0.969 (Figure 4). This indicates that XGB can effectively help predict the prognosis of SICH patients.

The “black box attribute” in ML refers to the challenge of understanding the internal decision-making process, even though accurate predictions can be generated based on given inputs. This lack of interpretability has limited the widespread application of ML methods in medical research (11, 15). SHAP, introduced by Lundberg et al. in 2017, is a method for explaining the predictions of ML models (18). Based on the concept of Shapley values from game theory, it decomposes the prediction result of a model into the contributions of each feature toward the prediction. SHAP helps medical researchers identify the most critical features for model predictions and explains the contribution of each feature to the outcome, thus aiding in understanding the basis of patient diagnostic results for subsequent analysis or therapeutic actions (19).

Similar to the ICH score proposed by Hemphill, our study found that the GCS score at admission, age, and hematoma volume are significant predictors of poor prognosis in patients with Intracerebral Hemorrhage (ICH). The GCS score, which evaluates a patient’s eye, verbal, and motor responses, has been extensively used since its introduction in 1974 to assess the severity of brain dysfunction (20). Our research indicates a significant correlation between poor prognosis in ICH patients and lower GCS scores. According to a prognosis model for hypertensive cerebral hemorrhage established by Ding et al., a hematoma volume of ≥25 mL and a GCS score of ≤12 were considered independent risk factors affecting patient prognosis (21). The ICH score system (ICHOP3) developed by Gupta et al., where a GCS score of 3–8 and a hematoma volume greater than 30 mL is valuable in predicting the functional state 3 months later (22), aligns with our findings where the average hematoma volume for patients with poor prognosis was 35 mL, compared to 7.3 mL for the good prognosis group. Typically, the larger the hematoma volume, the more significant the compression and worsening of the pathophysiological processes, leading to higher intracranial pressure, a critical factor in poor prognosis (23).

Moreover, Law et al.’s study suggests that rapid expansion of the hematoma is a significant reason for the deterioration of neurological functions in ICH patients, with those experiencing neurological deterioration showing more significant increases in hematoma volume (24). However, our study did not include hematoma expansion due to the inconsistency in the timing of measuring hematoma volume changes based on imaging data, making it difficult to establish a temporal relationship between hematoma volume change and prognosis. Additionally, advanced age is closely related to poor prognosis and higher mortality rates in patients with SICH. The increase in age implies changes in brain structure, such as cerebral amyloid angiopathy and brain atrophy, which may lead to increased local bleeding and more severe neurological damage (25, 26). Moreover, elderly patients often delay seeking emergency treatment due to physical limitations or lack of stroke awareness, further delaying disease management (27).

We found that patients with poor prognosis had higher baseline blood pressure and a significantly greater change in blood pressure from the emergency room to hospital admission. Elevated blood pressure changes are closely related to poor prognosis in patients with SICH. Li et al.’s study found that patients with rapid bleeding (intracerebral hemorrhage volume/onset to CT time > 5 mL/h) who received intensive blood pressure reduction treatment within 2 h after clinical symptoms appeared to significantly improve functional independence (28). Thus, clinical guidelines recommend early blood pressure reduction treatment upon emergency admission to prevent hematoma expansion and improve secondary outcomes (29). Coagulopathy is considered one of the most critical risk factors for SICH, often accompanied by the expansion of intracerebral hematomas. Early detection and intervention of acute coagulation dysfunction can significantly reduce mortality and improve prognosis (30, 31). Critically ill patients can develop stress-induced hyperglycemia early in the disease, and many studies have found high blood glucose levels closely related to poor prognosis after cerebral hemorrhage. Elevated blood glucose concentrations can increase vascular fragility and induce vascular rupture, leading to further expansion of the hematoma (32, 33). The mechanism might involve insulin resistance triggered by cerebral hemorrhage, where persistent high blood glucose promotes endothelial damage, combined with the compressive effects of the hematoma, adversely affecting local blood circulation and cerebral blood flow perfusion, causing the brain to remain in a state of ischemia and hypoxia for an extended period. This induces the accumulation of toxic metabolites, exacerbating cerebral edema and neuronal cell dysfunction (34, 35).

We found that platelet count did not show a significant difference in traditional statistical analysis, but it was important in the SHAP value analysis. This could be due to the interaction effect between platelets and other variables. ML models can capture these relationships, and their comprehensive consideration of multiple variables makes certain features important in the overall model, which is further reflected by SHAP values. Secondly, when the distribution of platelet counts overlaps significantly between the two groups, it may not show a significant difference statistically. However, platelet count may still contain valuable information across the entire dataset, which could be identified by the ML model. SHAP values quantify each feature’s contribution to the prediction output, indicating that even if a feature appears non-significant in traditional statistical tests, it may still play an important role in the model’s complex decision-making path. In clinical practice, the ICH score is widely used to predict 30-day mortality after ICH. As our results show, the XGB-based model significantly outperformed the ICH score in predicting the functional outcomes of patients. We believe that using the ICH scoring system for rapid assessment in emergency settings is necessary. At the same time, incorporating ML methods for detailed prognostic prediction, where feasible, can provide dual protection for patients. More importantly, continuous validation and updating of ML models with clinical data are crucial to ensure their predictive performance and adaptability, while interpretability analysis can enhance their clinical acceptability.

Based on the model’s predictions, we can optimize personalized treatment plans. By establishing a fast-track green channel, we can optimize the allocation of emergency department resources, prioritizing patients predicted to have poor functional prognoses. This can enhance emergency response efficiency and treatment outcomes. Intensive care unit resources can be preferentially allocated to high-risk patients. Additionally, rehabilitation resources can be proactively planned to meet the needs of specific patient groups. In the future, we plan to integrate the predictive results into the hospital admission process, conducting real-time risk assessments by combining imaging and laboratory data. During subsequent treatment, the model’s predictions will be continuously updated to help doctors dynamically adjust treatment strategies. Furthermore, we aim to embed the model into the existing electronic health record system, allowing doctors to view the prediction results and their explanatory analysis directly within the patient’s medical record interface.

When applying SHAP-based ML models in hospital clinical practice, in addition to the model’s predictive performance and interpretability, it is essential to consider the potential challenges and costs involved in its real-world implementation. First, for the prognosis prediction of SICH patients, hospital differences in medical record data formats and the laboratory methods employed can create difficulties in data integration. Hospitals need to standardize data formats and ensure the quality of clinical and imaging data to enable effective training and validation. Secondly, the development, training, and ongoing updates of the model require dedicated data science teams and computational resources. Additionally, ML models need to be regularly updated and retrained to adapt to new clinical data and evolving healthcare environments, which necessitates continuous financial investment. Furthermore, to enhance doctors’ trust and acceptance, hospitals should provide training on ML and SHAP analysis, which will involve time costs and investment in training resources. Finally, to meet data privacy and ethical requirements, hospitals may need to allocate additional resources for data encryption, anonymization, and compliance with relevant regulations. The development and use of the model may also require approval from an ethics review board. These multifaceted challenges require hospitals and research institutions to balance technological innovation with practical feasibility to ensure the model can be smoothly implemented and remain effective in real-world settings.

In the prognosis prediction of SICH patients, the interpretability based on SHAP analysis can significantly enhance doctors’ trust and acceptance of ML models. SHAP analysis helps clinicians identify the key factors influencing each patient’s prognosis, allowing for more attention to be given to treatment decisions. This individualized explanation enhances doctors’ trust in the model, as they can use these insights for more refined interventions and dynamically adjust treatment strategies based on the patient’s specific situation. Moreover, interpretability analysis provides a common understanding platform for doctors from different specialties (such as neurologists, radiologists, and emergency physicians), fostering interdisciplinary collaboration and improving overall treatment outcomes. Finally, when training doctors to use ML tools, the intuitive explanations provided by SHAP analysis can help doctors better understand the context and foundations of model predictions. This lowers the technical barrier and helps doctors quickly master how to use these tools in daily clinical decision-making, thereby improving model acceptance. Therefore, by enhancing the application of interpretability analysis, healthcare institutions can better integrate ML models into clinical practice, offering more precise and efficient medical services to patients.

However, our study does have certain limitations. First, although the patient data in this study were collected from multiple centers, it only included patients from three hospitals in China. There are significant differences in medical resources, technical expertise, and treatment processes across different regions in China. Regional preferences for conservative treatment or early surgical intervention can directly affect patients’ recovery speed and prognosis, potentially impacting the generalizability of our prediction model. Second, regional health disparities, such as differences in lifestyle, dietary habits, genetic background, and socioeconomic status, may lead to varying pathological mechanisms and clinical manifestations, which in turn influence treatment responses and short-term outcomes. Therefore, while our prediction model demonstrates good predictive performance for patients in specific regions of China, it may not yield the same results in other regions. Future studies should incorporate data from diverse regions and healthcare systems to validate the generalizability of the model and improve its global applicability. Third, due to the retrospective nature of this study, some relevant variables after admission, such as changes in neuroimaging and rehabilitation management, were not included in the research. Even though the model has achieved good predictive effects, it still cannot entirely replace judgment based on clinical experience. Therefore, in future studies, we still need to make comprehensive decisions based on individual patient situations and clinical contexts, continuously optimizing and improving ML models through the accumulation of clinical experience.

## 5 Conclusion

In this study, we developed an ML model based on XGB to predict the prognosis of patients with ICH and used SHAP to explain the model’s predictive results. The study found that the GCS, changes in SBP and DBP, hematoma volume, blood glucose levels, age, and coagulation function are key factors in identifying patients with poor prognosis. The ML model constructed in this study performed well in prediction. Combining the XGB algorithm with SHAP values provides a clear explanation for risk prediction and can offer valuable information for the clinical management of ICH patients, assisting doctors in making appropriate medical decisions.

## Data Availability

The raw data supporting the conclusions of this article will be made available by the authors, without undue reservation.
